# Psychiatric sequelae of thromboangiitis obliterans: a case report and review of the literature 

**DOI:** 10.1186/s13256-022-03694-z

**Published:** 2023-01-30

**Authors:** Mahmoud A. Awara, Laura M. Downing, Manal O. Elnenaei

**Affiliations:** 1 Department of Psychiatry, Dalhousie University, Medical School, Halifax, Canada; 2 The College of Physicians and Surgeons of Nova Scotia, Halifax, Canada; 3 The Royal College of Psychiatrists, London, UK; 4 Department of Pathology and Laboratory Medicine, Dalhousie University, Medical School, Halifax, Canada; 5 The Royal College of Pathologists, London, UK

**Keywords:** Treatment resistant organic psychosis, Thromboangiitis obliterans inducing psychosis, Case report, Buerger's disease and schizophrenia-like-psychosis

## Abstract

**Background:**

Peripheral manifestations secondary to progressive vascular occlusions are characteristic of the rare condition termed thromboangiitis obliternas (TAO) or Buerger’s disease. The central manifestations of this disease are however poorly characterized, particularly those of psychiatric nature, and their prevalence is largely unknown. Speculations have been made around the polymorphic nature and triggers of observed psychopathology in TAO; much however remains to be unraveled in this area.

**Case presentation:**

We present the case of a 33-year-old Caucasian male who developed first episode of psychosis at the age of 29 years. There was no history of previous mental illness either in the patient, or in any of his family members. He had been a long- term heavy smoker and was experiencing progressive lower limb claudication since the age of 22 years; however, all inflammatory, autoimmune and atherosclerotic markers were negative. His psychosis was characterized by retention of a warm affect, and despite some amelioration, was generally resistant to a fair trial of several anti-psychotic medications including Clozapine.

**Conclusion:**

The pathophysiology of psychosis secondary to Buerger’s is not yet well characterized which adds to the complexity of managing these cases. Recognizing that cerebral manifestations of this disease may evolve several years after the onset of peripheral thromboangiitic features is important for following the natural history and considering measures that may reduce the burden of illness.

## Background

We present a young male patient who developed treatment resistant psychosis secondary to central nervous system manifestations of Buerger’s disease.

Organic/secondary psychosis can mimic functional/primary psychotic conditions e.g. schizophrenia and this may delay early recognition and timely management of patients with such conditions.

## Case presentation

### Psychiatric history

We describe the case of a 33-year-old Caucasian male patient who developed his first episode of psychosis in 2017 at the age of 29 years. This resulted in a psychiatric admission that lasted two months but led to no significant improvement of his psychotic symptoms. Prior to this admission he had no previous mental health issues or contact with psychiatric services.

Over the two-month in-patient period he was trialed on an antipsychotic, Paliperidone Palmitate long acting injection, that was later switched to oral Aripirazole as the former caused excessive sedation. He was discharged on the latter but remained psychotic in the community, hence, his treatment was switched to Lurasidone 80 mg for three months, yet his symptoms were still unresolved. He was subsequently admitted to an inpatient rehabilitation unit for one year.

The patient reported tobacco smoking of 20–25 cigarettes daily and 4–5 g of cannabis every 2–3 days but denied other substance misuse. Despite the service’s endeavours to encourage him to stop smoking tobacco and cannabis, the patient declined changing his habit. During his lengthy rehab admission, his Lurasidone dose was increased from 80 to 160 mg but he continued to experience polymorphic psychosis in the form of persecutory, grandiose delusions, thought broadcast and delusion of control. In addition, he experienced auditory hallucinations to which he was attending.

Due to the nature of his treatment resistant psychotic symptoms, he was started on Clozapine and the dose was gradually titrated to 450 mg daily. His psychotic symptoms remained active but were less disabling as he stopped responding to the auditory hallucinations and started to actively engage in rehabilitation programming,

Following a dose reduction of Clozapine to 400 mg, in response to the patient’s request, he became noticeably psychotic, responding to unseen stimuli. Attempts to increase the Clozapine dose back to 450 mg was consistently declined by the patient, and he was eventually discharged to the community on 400 mg Clozapine. Of note, during his admission to rehab he was not using illicit drugs and managed to quit his long-term habit of tobacco smoking; thus, his Clozapine dosage was within the target dose for his gender and non-smoker status.

Upon his discharge he was referred to the community rehab team and on initial assessment he reported severe psychotic symptoms in the form of delusion of control via a microchip that he believed had been implanted in his brain by nurses. He reported that via this microchip nurses were able to insert, withdraw and broadcast his thoughts, as well as determine his thoughts a few seconds prior to him experiencing them. In addition, he reported constantly hearing nurses’ voices, in an extracampine manner, from a different city. Despite the severity of his reported psychosis he requested reduction of his Clozapine; but he later disclosed that he had unilaterally reduced the dose to 200 mg, which could explain the worsening of his symptoms.

The patient then agreed to consider alternative antipsychotic treatment that included oral Risperidone; but his non-compliance with oral treatment became an issue. Depot medication of Zuclopenthixol decanoate IM was then trialed; this initially dampened his psychotic experience, but later caused severe akathisia and agitation that was unresponsive to a beta blocker and Diazepam in combination. This triggered a third admission to acute psychiatry services where his psychotropic medication was switched to Olanzapine.

He continued to be treated on Olanzapine 20 mg daily in the community, which as such did not ameliorate his psychotic symptoms but reduced their consequential effect on his mental status. In his most recent mental state examination in March 2021, he disclosed complex systematized delusions, describing a state of ‘over-ride’ by God, of his self-control, directing him to do or not to do certain things e.g., smoking, working, etc. He also believed he is being controlled by the government via a microchip implanted in his brain and reported constantly hearing four benevolent voices ‘keeping him company’. He currently lives in a state of harmony with his psychosis and reported no distress and his affect became indifferent.

Personal and family history for this patient were both unremarkable. The patient disclosed that he started to use recreational drugs at the age of 17; he was smoking 1–2 joints of Cannabis daily but stopped using that during his rehab admission. He experimented on LSD once at the age of 17. He reported a single use of MDMA in his early 20s. He also used Cocaine between the age of 22 and 24 of 0.5–1 g a few days weekly. He denied excessive alcohol drinking or any binge-drinking.

### Medical history

The patient’s significant medical history goes back to when he was 22 years of age, i.e. 7 years prior to his first episode psychosis. He started to complain then of progressive claudication in his right leg over 1–3 years before he experienced pain at rest in this leg. When investigated by CT angiography in 2011, his right superficial femoral artery was found to be occluded (mid-thigh level) along with the right popliteal artery (mid-calf level). The other remarkable medical finding was that he had congenital deafness seemingly inherited from his paternal grandfather.

### Rheumatology and connective tissue disease consult in 2011

Primary inflammatory vasculopathy and primary autoimmune processes were ruled out. An extensive battery of autoimmune and inflammatory markers were negative that included lupus, anticardiolipin antibodies, homocysteine and antiphospholipid antibody.

### Cardiology and vascular surgery consult in 2011

Hereditary aneurysm syndrome and other vasculopathies were also ruled out via an extensive battery of investigations, and there was no family history of mid-vessel occlusion; hence, early Buerger’s syndrome was suspected. Subsequently, in 2011 he had a successful right femoral bypass using a reverse right saphenous vein graft. In 2012 the graft became occluded, and the patient was admitted for thrombolysis. Between 2012 and 2015, the same was repeated on three occasions and was ascribed to heavy smoking. He, therefore, had three further admissions to vascular surgery for graft occlusion that was associated with severe claudication. This was treated by thrombolysis, catheter insertion for tPA (tissue Plasminogen Activator) infusion to dissolve the thrombosis and he was maintained on conservative treatment in the form of Pentoxifylline 400 mg SR BID; Clopidogrel 75 mg OD; Rosuvastatin 10 mg OD; and Omeprazole 20 mg OD.

The deterioration of his vascular condition was attributed to heavy smoking compounding the manifestations of thromboangiitis obliterans.

## Special investigations

### Laboratory investigations (between 2011 and 2021)

Apart from a low serum urea between January and February 2011 and a transiently high ammonia in October 2019—all other blood test results for this patient did not show any abnormality. These included the following tests to exclude aetiologies for his vascular condition: lipid profile (average total cholesterol: 3.3 mmol/L, LDL: 2 mmol/L), ANA, rheumatoid factor and cryoglobulin.

### CT abdominal and bifemoral Scan in 2012 (Fig. 1A, B)

**Fig. 1:**
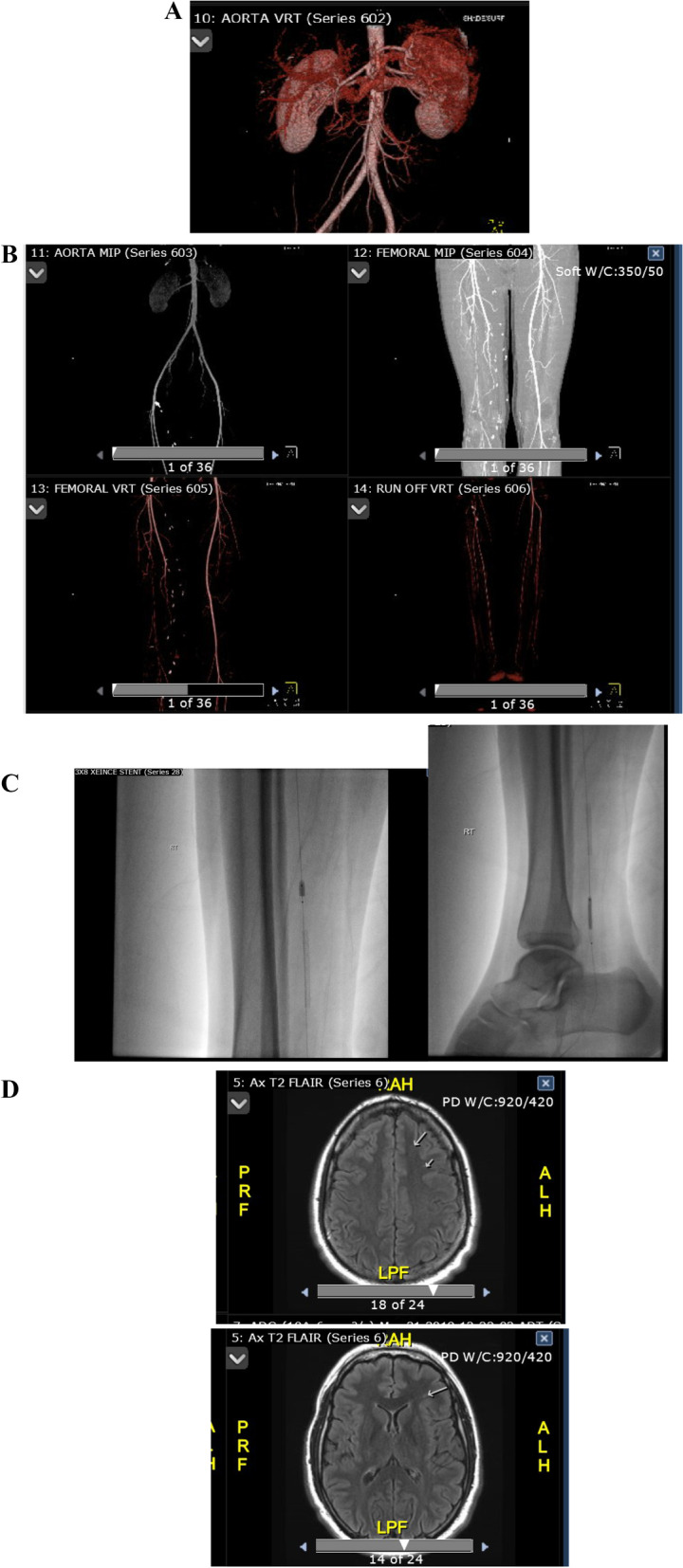
**A** Abdominal CT Scan Demonstrating Patent Abdominal Aorta.** B** Patent aortic bifurcation but right superficial femoral artery (SFA) was occluded along with the venous graft connecting the distal SFA to the tibioperoneal trunk.** C** Successful subintimal recanalization and angioplasty was performed of the right distal posterior tibial artery occlusion as well as the proximal peroneal artery occlusion.** D** MRI brain scan demonstrating a few tiny left frontal subcortical and periventricular T2/FLAIR hyperintense nonspecific foci

This scan demonstrated normal abdominal aorta, and other arteries were patent but right superficial femoral artery (SFA) was occluded along with the venous graft connecting the distal SFA to the tibioperoneal trunk.

### Interventional radiology in 2013 (Fig. 1C)

Successful subintimal recanalization and angioplasty was performed of the right distal posterior tibial artery occlusion as well as the proximal peroneal artery occlusion.

### Treadmill exercise evaluation in 2017

This test was ended within five minutes due to onset of pain in the right calf muscle at three minutes and thirty seconds. The test demonstrated severe ischemia of the right leg at ankle/pedal level and mild ischemia of the left leg post exercise.

The test also demonstrated disease progression of both legs when compared with a previous test conducted in 2014.

### MRI brain scan in 2019 (Fig. 1D)

No significant structural abnormality was identified; however, a few tiny left frontal subcortical and periventricular T2/FLAIR hyperintense nonspecific foci were noted.

### Electroencephalogram (EEG) in 2019

EEG recording demonstrated generalised grade 2 dysrhythmia that was ascribed to psychotropic medications. There were no focal or lateralizing features or epileptiform discharges identified.

### Positron emission tomography (PET) brain scan in 2021

There was some mild generalized hypometabolism involving temporal, parietal and frontal regions with mild accentuation of the sensorimotor strip. It may relate to long-term medication treatment. No definite focal areas of hypometabolism were identified within this scan. There was no evidence of lateralization of activity in the temporal lobes. There were no definite metabolic findings to help localize an epileptic seizure focus in this patient.

### Buerger’s disease: epidemiology, etiology and pathophysiology

Peripheral manifestations secondary to progressive vascular occlusions are characteristic of the rare condition termed Thromboangiitis Obliternas (TAO) or Buerger’s disease. The central manifestations of this disease are however poorly characterized, particularly those of psychiatric nature, and their prevalence is largely unknown. Speculations have been made around the polymorphic nature and triggers of observed psychopathology in TAO; much however remains to be unraveled in this area.

Critical limb ischemia (CLI) was first described by Felix von Winiwarter in 1879 [1] when he published his first case report of a patient with this condition [2–4]. In 1908 Leo Buerger described an unusual form of progressive vascular occlusion in Polish and Russian immigrants who underwent amputation for CLI [5]. Buerger called the disease ‘thromboangiitis obliterans’ (TAO) to distinguish it from arteriosclerosis obliterans (ASO).

The estimated worldwide prevalence of Buerger’s disease is 5–12 per 100,000 population per year, and it most commonly affects patients from the Middle East, Asia, the Mediterranean and Eastern Europe. Although it is rarely reported, TAO accounts for a substantial proportion of peripheral vascular disease ranging from 66% in Korea and India [2], 0.75% in America but up to 5.6% in Western Europe with a higher rate among Mediterranean populations of 39% [6]. TAO is more common in younger men and the disease affects small and medium sized vessels distally before it moves to proximal arteries [7].

The aetiology of Buerger’s disease is unknown but the disease activity is strongly associated with tobacco smoking beyond any debate. Other potential etiological factors are genetic predisposition, immune mediated response, hyper-coagulable state and oral infection-inflammatory-pathway [8].

**Fig. 2:**
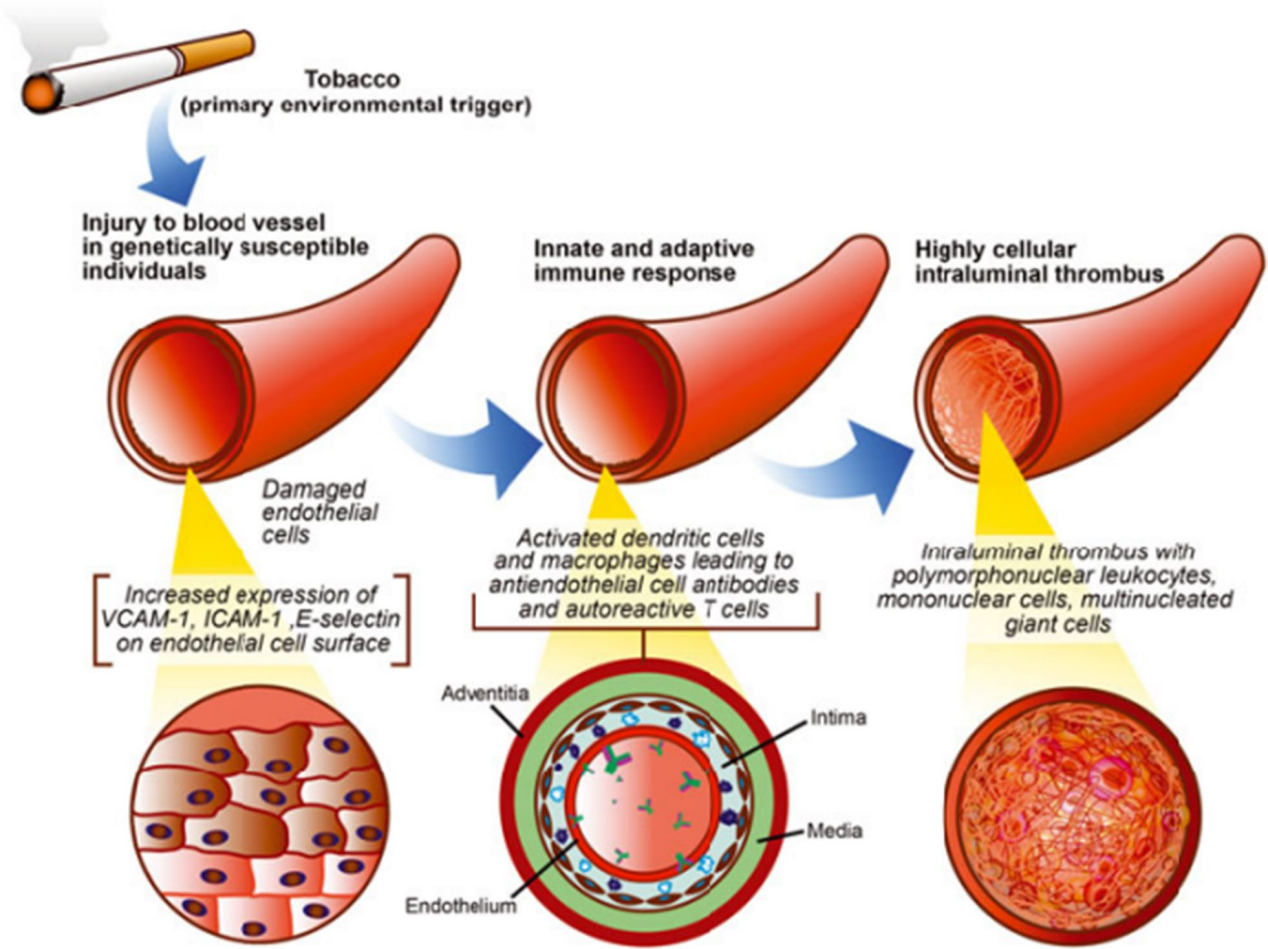
Pathogenesis of Buerger’s Disease (thromboangiitis obliterans). Reproduced with Permission from Dr. Cooper and Annals of the New York Academy of Sciences [9], (Order License ID 1311888-1, ISSN 1749-6632)

Buerger’s disease is characterized by segmental inflammatory cell infiltration of the vessel wall and subsequent arterial or venous thrombotic occlusions. There is growing evidence that the primary event is related to endothelial cell damage induced by an unidentified antigen, notably tobacco glycoproteins [6]. Also there are possible links between tobacco exposure and loss of tolerance for vascular tissues [9]. This autoimmune inflammatory process is limited to the arterial intima and mediated by T cell infiltration. The resulting arterial occlusion is characterized by well-preserved architecture of the vascular walls which is pathognomonic for TAO and is in contrast to atherosclerosis and systemic vasculitis [6, 8, 9]. (Fig. 2).

### Common manifestations of Buerger’s disease

The most common symptoms are claudication affecting the arch or lower calf due to infrapopliteal occlusion. Other early symptoms include cold insensitivity, burning pain in the feet and hands, cyanosis, skin atrophy, migratory superficial thrombophlebitis, Raynaud’s phenomenon and reduced hair growth. Less commonly, Buerger’s disease may affect visceral, cerebral, coronary, internal thoracic vessels and multi-system involvement [6].

Multi-organ involvement can be troublesome as occlusion of visceral vessels are associated with a myriad of life-threatening sequelae e.g. intestinal perforation and mesenteric infarction [10]. Rarely however, TAO may affect a coronary artery and present as acute myocardial infarction.

Non-erosive arthritis symptoms are reported commonly in the wrists and knees in 12% of cases with TAO, 10 years on average before the occlusive phase. Large joints are particularly affected, symptoms are of a migratory nature and are accompanied by signs of local inflammation [11].

Central nervous manifestations have been reported in patients with TAO. In their review, Hausner and Allan [12] found 11 cases of cerebral manifestations in a group of 500 patients with TAO at the Mayo clinic with an incidence rate of 2.2%. They reported that the commonest cerebral symptoms of TAO were transient hemianopia, recurrent hemiplegia, transient confusion, disorientation, aphasia, loss of memory and epileptiform convulsions. The peripheral vascular manifestations of TAO preceded the neurological symptoms in nine of these cases by 5 months to 20 years. However, in three of the TAO cases, the neurological manifestations preceded the peripheral vascular manifestations by 1 to 14 years, which may cause a diagnostic quandary.

### Buerger’s disease and psychiatric manifestations

Little is known about the prevalence of psychiatric manifestations of TAO. Perk *et al*. [13], reported that a patient who was admitted for 16 months, had presented with a wide range of symptoms that encompass primary diagnoses of schizophrenia, mania, manic-depressive psychosis, epileptic psychosis that concomitantly occurred with vascular manifestations of TAO. He suggested that the mechanism of vascular changes may take one of two trajectories: (1) a slow and progressive occlusion of the vessels affected, (2) paroxysmal functional disturbance induced by the physical change of the vessel wall. It is believed that the prolongation of the functional disturbance can lead to irreversibility that is applicable to the brain blood vessels and hence, could trigger polymorphic psychopathology of a wide-spectrum psychiatric nosology.

Diagnostically, our reported case satisfies Shionoya criteria for Buerger’s disease which comprises: onset before age 50; smoking history; infrapopliteal arterial occlusions, upper limb involvement or phlebitis migrants; absence of atherosclerotic risk factors other than smoking [14].

## Discussion

The reported patient developed first episode psychosis at age 29 which is 4 years later than the expected second peak of schizophrenia in males. Even though his psychosis was of a relatively late onset and he has no family history of psychosis which should carry a good prognostic indication, his condition was treatment resistant from the outset and he was unresponsive to several antipsychotic medications including Clozapine.

In addition, this patient experienced peripheral vascular manifestations of Buerger’s disease seven  years prior to his psychiatric presentation. This highlights that his psychosis is a sequela of cerebral manifestations secondary to Buerger’s disease, that occurs years after peripheral vascular manifestations, as reported in the majority of cases by Hausner and Allan (1938).

PET brain scan was performed to examine the presence of cerebrovascular sequalae of TAO e.g. epileptic focus [12, 13] that could induce psychosis at a nonconvulsive level. The findings, however, were rather of non-specific mild generalized hypometabolism in temporal, parietal and frontal regions with mild accentuation of the sensorimotor strip with no localization for epileptic focus. This non-specificity in the PET scan findings could be explained by the protean nature of the cerebrovascular manifestations of TAO [15] and the lack of PET scan studies to highlight markers for these manifestations.

The patient’s brain MRI scan had shown hyperintense nonspecific tiny foci scattered in the left frontal subcortical and periventricular regions that could represent cerebrovascular changes of Buerger’s disease.

EEG was remarkable for grade 2 dysrhythmia with no epileptiform spikes noted; we wondered, however, as to whether this dysrhythmia could be merely ascribed to psychotropic medications or to early changes related to cerebral manifestations of Buerger’s disease. No data, however, is available about the pathognomonic EEG changes in TAO.

Bozikas *et al.* [16] described in their case report a 55-year-old patient who was formally admitted for persecutory and reference delusions that was associated with auditory hallucinations and psychomotor agitation secondary to fears of being poisoned. The patient’s psychiatric history started at the age of 50, which was 20 years following the onset of peripheral vascular manifestations of TAO that culminated in gangrene and finger and toe amputations. This patient had also been a heavy smoker with no family history of mental illness. In his discussion, Bozikas argued that the patient’s psychosis could be caused by the cerebral manifestations of TAO causing deep periventricular white matter lesions.

Harten *et al*. [17] reported multiple organ manifestations in TAO in a 23-year-old male smoker that resulted in myocardial, splenic and cerebral infarctions, pulmonary embolisms, and intestinal ischemia. This may indicate the versatility of the disease that can impact several organs at the same time in a young patient.

Zülch [4] reported a 31-year-old man who suddenly died from a massive haemorrhage at an atypical site in the frontal lobe. The post-mortem autopsy revealed whitish, bloodless, solid and shrunken terminal arteries of the cortex; granular atrophy of the cortex, and vigorously filled meningeal arterial rings and cerebellar arteries. Zülch noted that these changes affected small cerebral and cerebellar arteries that were occluded by loose connective tissues, but otherwise these vessels were healthy with intact intima which is characteristic of TAO.

Little is known about the prevalence of cerebral manifestations of TAO, given that the incidence rate of vascular manifestations significantly differs geographically and ranges between 0.75% in America to 66% in Korea and India. The main identified risk factor, however, for TAO is tobacco smoking in the predisposed population.

Whether the psychiatric manifestations of our reported patient are incidental or directly connected to TAO may be difficult to answer with certainty. However, our reported case has an established diagnosis of TAO which preceded his psychiatric symptoms by seven years. Even though this patient developed his first episode psychosis at the age of 29 (relatively late onset in males), he was treatment resistant from the outset and did not respond to a fair trial on Clozapine along with several antipsychotic medications. In addition, the patient maintained a warm affect and did not develop negative symptoms of schizophrenia which would be expected in treatment resistant cases, substantiating that his psychosis could be organic in nature.

Furthermore, the patient’s brain PET and MRI scans had shown non-specific generalized hypometabolism and a few tiny left frontal subcortical and periventricular hyperintense foci. Whether these emulate changes noted by Zülch [4] in cases of post-mortem cerebral changes secondary to TAO would be difficult to ascertain in our patient without histopathological examination.

The rarity and protean nature of the cerebrovascular manifestations of TAO that may either precede or follow the peripheral vascular manifestations of the illness by years, could confound the clinical picture. Moreover, without extensive histopathological examination of cerebral vessels, which is untenable in a living patient, the diagnostic challenges are augmented.

## Conclusion

Our conclusion about the organic nature of this patient’s psychosis is based on that his clinical presentation does not follow the natural history of primary psychosis. The cerebral manifestations of TAO remain to be in the realm of case reports and clinical observations. We hope by sharing this case report with the scientific community to raise awareness that psychiatric manifestations in TAO may evolve many years after initial diagnosis and shed light on the complexity of managing these cases. There may also be an opportunity for life-style changes that could be instigated early on prior to central affection, to reduce the burden of disease. We also hope that as more sophisticated techniques evolve, direct linking of cerebrovascular changes of TAO with its psychopathological manifestations, would be possible.

## Data Availability

Not applicable.

## References

[1] von Winiwarter F (1879). Ueber eine eigenthümliche Form von Endarteriitis und Endophlebitis mit Gangrän des Fusses. Arch Klin Chir.

[2] Olin JW (2000). Thromboangiitis obliterans (Buerger's disease). N Engl J Med..

[3] Shionoya S (1990). Buerger's disease: pathology, diagnosis and treatment.

[4] Zülch KJ (1969). The cerebral form of von Winiwarter-Buerger's disease: does it exist?. Angiology.

[5] Buerger L (1908). Thrombo-angiitis obliterans: a study of the vascular lesions leading to presenile spontaneous gangrene. Am J Med Sci.

[6] Akar AR, İnan MB, Baran Ç (2016). Thromboangiitis obliterans. Curr Treat Options Rheum.

[7] Olin JW, Shih A (2006). Thromboangiitis obliterans (Buerger’s disease). Curr Opin Rheumatol.

[8] Iwai T, Inoue Y, Umeda M (2005). Oral bacteria in the occluded arteries of patients with Buerger disease. J Vasc Surg.

[9] Ketha P, Cooper S (2013). The role of autoimmunity in thromboangiitis obliterans (Buerger's disease). Ann New York Acad Sci..

[10] Sauvaget F, Debray M, De Sigalony J-PH (1996). Colonic ischemia reveals thromboangiitis obliterans (Buerger’s disease). Gastroenterology.

[11] Vijayakumar A, Tiwari R, Prabhuswamy VK. Thromboangiitis obliterans (Buerger’s Disease)—current practices. Int J Inflamm. 2013; Article ID 156905, 9. 10.1155/2013/156905. 10.1155/2013/156905 PMC3786473 24102033

[12] Hausner E, Allen EV. Ann Int Med. 1938; (12): 845–852.

[13] Perk D (1947). Cerebral symptoms in thrombo-angiitis obliterans. J Meteorol Soc Jpn.

[14] Shionoya S (1998). Diagnostic criteria of Buerger’s disease. Int J Cardiol.

[15] Lippmann HI. Cerebrovascular thrombosis in patients with Buerger's disease. Circulation. 1952; (5.5): 680–692. 10.1161/01.cir.5.5.680 14926052

[16] Bozikas VP, Vlaikidis N, Petrikis P, Kourtis A, Karavatos A (2001). Schizophrenic-like symptoms in a patient with thrombo-angiitis obliterans (Winiwarter-Buerger's disease). Int J Psychiatry Med.

[17] Harten P, Müller-Huelsbeck S, Regensburger D, Loeffler H (1996). Multiple organ manifestations in thromboangiitis obliterans (Buerger's disease): a case report. Angiology.

